# Prognostic implications of left ventricular mass-geometry in patients with no or nonobstructive coronary artery disease

**DOI:** 10.1186/s12872-021-02005-6

**Published:** 2021-04-15

**Authors:** You-Jung Choi, Jun-Bean Park, Chan Soon Park, Inchang Hwang, Yeonyee E. Yoon, Seung-Pyo Lee, Hyung-Kwan Kim, Yong-Jin Kim, Goo-Yeong Cho, Dae-Won Sohn

**Affiliations:** 1 Division of Cardiology, Department of Internal Medicine/Cardiovascular Center, Seoul National University Hospital, 101 Daehak-ro, Jongno-gu, Seoul, 03080 Republic of Korea; 2 Department of Internal Medicine, Seoul National University College of Medicine, Seoul National University, Seoul, Republic of Korea; 3 Graduated School of Medical Science and Engineering, Korea Advanced Institute of Science and Technology, Daejeon, Republic of Korea; 4 Cardiovascular Center, Seoul National University Bundang Hospital, Seongnam, Gyeonggi-do Republic of Korea

**Keywords:** Left ventricular remodeling, Coronary artery disease, Coronary computed tomography angiography, Mortality

## Abstract

**Background:**

Coronary computed tomography angiography (CCTA) is widely used as a first-line noninvasive modality that frequently exhibits no or nonobstructive coronary artery disease (CAD) in clinical practice, along with abnormal left ventricular (LV) geometry on echocardiography. However, the combined prognostic value of these findings has not been well elucidated. Therefore, we aimed to evaluate the prognostic implications of abnormal LV geometry in individuals with no or nonobstructive CAD.

**Methods:**

A total of 5806 subjects with no CAD or nonobstructive CAD (luminal narrowing < 50%) on CCTA were included in the study. The major exclusion criteria were structural heart disease and a history of myocardial infarction or coronary revascularization. Abnormal LV geometry on echocardiography was defined as LV mass index > 95 g/m^2^ in women and > 115 g/m^2^ in men, and/or relative wall thickness > 0.42. The primary outcome was all-cause mortality.

**Results:**

A total of 5803 subjects without significant obstructive CAD (age, 56.6 ± 8.87 years; men, 3884 [66.9%]). Of them, 4045 (69.7%) subjects had normal LV geometry and 1758 (30.3%) had abnormal LV geometry respectively. During a mean follow-up of 6.2 ± 1.48 years, 84 (1.44%) subjects died in the study population. Of these, 56 subjects were from the normal LV geometry group (1.24%) and 28 were from the abnormal LV geometry group (2.32%). Subjects with abnormal LV geometry had significantly worse survival rates (log-rank, *p* < 0.001). After adjustment for confounding factors, abnormal LV geometry was an independent predictor of all-cause mortality (adjusted hazard ratio, 1.64; 95% confidence interval, 1.04–2.58; *p* = 0.034). Moreover, abnormal LV geometry was significantly worse in survival when classified as those with no CAD (log-rank, *p* = 0.024) and nonobstructive CAD (Log-rank, *p* < 0.001).

**Conclusions:**

Abnormal LV geometry portends a worse prognosis in subjects with no or nonobstructive CAD. These findings suggest that LV geometry assessment can help improve the stratification of individuals with these CCTA findings.

**Supplementary Information:**

The online version contains supplementary material available at 10.1186/s12872-021-02005-6.

## Background

The emergence of coronary computed tomography angiography (CCTA) as a noninvasive imaging modality has made it possible to diagnose coronary artery disease (CAD) with excellent sensitivity (90–95%) and negative predictive value (93%–98%) [1]. In addition to these advantages, CCTA has been widely used to detect or exclude significant CAD, serving as a reliable gatekeeper for invasive coronary angiography. Consequently, in clinical practice, no CAD or nonobstructive CAD is frequently encountered on CCTA.

In previous studies, the prognostic importance of nonobstructive CAD has been substantiated already [2, 3]. Indeed, subsequent aggressive preventive interventions have been emphasized for patients with nonobstructive CAD than those without CAD [4, 5]. A recent study reported that nonobstructive CAD incidentally found on CCTA in the emergency department increases the likelihood of statin prescription [6]. The CCTA-based assessment of plaque size and its composition has recently gained particular attention as an imaging method to improve risk stratification and ultimately further statin allocation since this technique seems to enable the risk prediction of cardiovascular disease events in patients with nonobstructive CAD [7]. However, due to technical challenges in the analysis of plaque characteristics, it has not become a part of real-world clinical practice, suggesting the need for more readily available tests on a routine clinical basis.

Left ventricular (LV) hypertrophy is a consequence of cardiac geometric adaptation in response to systemic hemodynamics and ventricular load [8]. Abnormal LV geometric patterns are associated with systolic and diastolic dysfunction [9] and, more importantly, are well-established predictors of cardiovascular morbidity and mortality in various populations [10–13]. However, there is a paucity of data to inform the prognostic value of abnormal LV geometry for no or nonobstructive CAD detected by CCTA. Therefore, the current study aimed to evaluate the prognostic implication of echocardiography-determined LV geometry in individuals without obstructive CAD confirmed by CCTA.

## Methods

### Study design and patients

This was an observational retrospective multicenter cohort study. We reviewed 12,956 consecutive subjects who underwent CCTA and echocardiography between 2002 and 2011 at the Seoul National University Hospital, Seoul National University Bundang Hospital, and Seoul National University Hospital Gangnam Center. We excluded individuals with significant LV systolic dysfunction (defined as ejection fraction < 40%), hypertrophic or infiltrative cardiomyopathy, severe valvular heart disease, and those with a history of previous myocardial infarction, coronary revascularization, or cardiac surgery.

A total of 5803 subjects were included in the final analysis, who had available echocardiographic data on the left ventricular mass index (LVMI) and relative wall thickness (RWT) and had no significant coronary artery stenosis (defined as luminal narrowing < 50%) on CCTA (Additional file 1: Fig. 1). The date of the final follow-up was March 18, 2016.

**Fig. 1:**
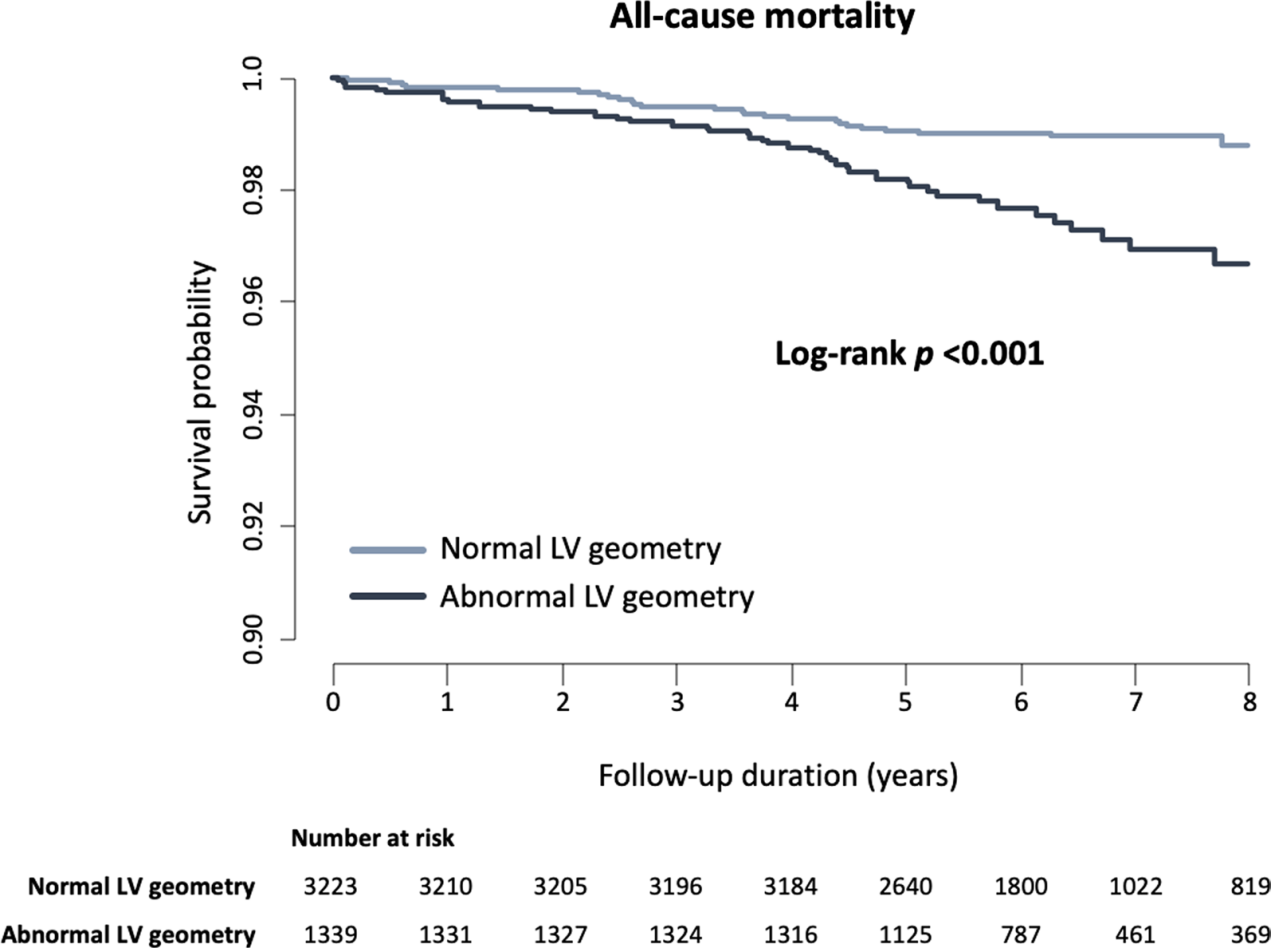
Kaplan–Meier survival curve for subjects with no or nonobstructive CAD on CCTA according to presence or absence of abnormal LV geometry. CAD, coronary artery disease; CCTA, Coronary computed tomography angiography; LV, left ventricular

The study protocol was approved by the Institutional Review Board of Seoul National University Hospital (IRB No. J-1511-025-715), and was performed in accordance with the Declaration of Helsinki. Written informed consent was waived owing to the retrospective and observational nature of this study.

### Image acquisition and analysis for CCTA

Electrocardiographic-gated CCTA images were acquired using a 64-slice multidetector scanner (SOMATOM Sensation 64, SOMATOM Definition, Siemens Medical Solutions, Forchheim, Germany; Brilliance 64, Philips Medical Systems, Best, The Netherlands). Before performing the scan, sublingual nitroglycerine (0.3 mg) was administered to provide transient coronary dilatation, and metoprolol (50 mg) was administered to subjects presenting with a heart rate of more than 60 beats per minute. The CCTA results were graded as none (luminal narrowing, 0%), nonobstructive (luminal narrowing, 1–49%), and obstructive (luminal narrowing ≥ 50%) respectively based on the severity of narrowing in any of the major epicardial coronary arteries.

### Echocardiographic analysis

All patients underwent comprehensive two-dimensional echocardiography within 3 months of initial CCTA. Hypertrophic alteration of the LV structure was quantified based on LVMI and RWT by echocardiography [14]. LVMI was estimated using a standard formula with LV cavity dimension and wall thickness at end-diastole and indexed to body surface areas. Linear internal measurement of the LV and its wall was performed in the parasternal long-axis view, and the values were obtained perpendicular to the long axis of the LV at or immediately below the level of the mitral valve leaflet tips [14]. LV mass index was calculated using the following equation:

1. LV mass = 0.8 × 1.04 × {[LV end-diastolic dimension (LVEDD) + interventricular septal wall thickness + LV posterior wall thickness]^3^ − LVEDD^3^} + 0.6
2. LV mass index = LV mass/body surface area

RWT was calculated as two times the posterior wall thickness divided by the LV diastolic diameter. Increased LVMI was defined as LVMI > 95 g/m^2^ in women and > 115 g/m^2^ in men, and the cutoff for abnormal RWT was > 0.42, in both women and men [14]. Normal LV geometry was defined as normal LVMI and RWT. Abnormal LV geometry was defined as a composite of concentric remodeling (normal LVMI and increased RWT), eccentric hypertrophy (increased LVMI and normal RWT), and concentric hypertrophy (increased LVMI and RWT).

### Laboratory tests

Using electronic medical records, we obtained laboratory information, including serum hemoglobin, total cholesterol, triglycerides, low-density lipoprotein cholesterol, high-density lipoprotein cholesterol, fasting blood glucose, creatinine, and estimated glomerular filtration rate (eGFR) accordingly for all the patients.

### Primary outcome

The primary outcome was all-cause mortality. Mortality data were obtained and verified via a centralized database of death records from the Korean Ministry of Security and Public Administration.

### Statistical analysis

Descriptive data were reported as mean ± standard deviation for continuous variables and as numbers and percentages for categorical variables. Shapiro–Wilk Normality test was performed to determine the distribution of data. We performed the Student’s *t*-test and Mann–Whitney U test for continuous variables according to the data distribution and Chi-square test for categorical variables for the comparison between normal and abnormal LV geometry groups. Further, a comparison of mean values between multiple groups was performed using one-way analysis of variance, and Kruskal–Wallis tests were used for continuous variables according to the data distribution, and the chi-squared test was performed for categorical variables, followed by a post-hoc comparison. Event-free survival analysis was performed using the Kaplan–Meier method and compared using the log-rank test. To investigate the association between LV geometry and all-cause mortality, hazard ratios (HRs) and 95% confidence intervals (CIs) were calculated using univariate and multivariate Cox proportional regression analyses. Multivariate analysis adjusting for statistically different baseline variables (age, sex, body mass index, serum hemoglobin, serum total cholesterol, and eGFR) was performed to provide independent effect estimates for abnormal LV geometry on the primary outcome.

A two-tailed *p* value of < 0.05, was considered statistically significant. All statistical analyses were performed using SPSS version 23 (IBM Corp, Chicago, IL, USA) and R programming version 3.2.4 (http://www.R-project.org; The R Foundation for Statistical Computing, Vienna, Austria).

## Results

### Baseline characteristics of the study population

A total of 5803 subjects without significant obstructive CAD consisted of 3884 (66.9%) men with a mean age of 56.6 ± 8.87 years (Table 1). Of them, 4045 (69.7%) subjects had normal LV geometry and 1758 (30.3%) had abnormal LV geometry. The subjects with normal LV geometry were younger than those with abnormal LV geometry (55.7 ± 8.68 vs. 58.5 ± 8.98 years, *p* < 0.001), and this was observed more frequently in men (69.6 vs. 65.9%, *p* < 0.001). There was no significant difference in the baseline laboratory results between the two groups, except for the decreased hemoglobin (14.4 ± 1.61 mg/dL in normal geometry vs. 14.6 ± 1.45 mg/dL in abnormal geometry, *p* < 0.001) and eGFR levels (77.2 ± 13.7 vs. 78.8 ± 13.3 mL/min/1.73 cm^2^, *p* < 0.001). With regard to echocardiographic parameters, the abnormal LV geometry group had increased LV wall thickness and chamber size, but also larger left atrial size (36.5 ± 4.95 mm vs. 38.0 ± 5.48 mm, *p* < 0.001), higher pulmonary artery systolic pressure (27.0 ± 4.63 vs. 28.1 ± 5.06 mmHg, *p* < 0.001), and a higher ratio of peak early transmitral inflow velocity to early diastolic velocity of the mitral annulus (E/e’) (9.34 ± 2.84 vs. 11.4 ± 2.05, *p* = 0.003) than the normal LV geometry group. The baseline characteristics according to the four LV geometric patterns are shown in Additional file 1: Table 1.

**Table 1:** Baseline characteristics

Values	Total (n = 5803)	Normal geometry (n = 4045)	Abnormal Geometry (n = 1758)	*P* value
Age, years	56.6 ± 8.87	55.7 ± 8.68	58.5 ± 8.98	< 0.001
Male sex, n (%)	3884 (66.9)	2816 (69.6)	1068 (65.8)	< 0.001
Body mass index, kg/m^2^	24.4 ± 2.85	24.3 ± 2.77	24.6 ± 2.99	< 0.001
Medications, n (%)				
Statins	258 (4.4)	176 (4.4)	82 (4.7)	0.644
ARB/ACEI	243 (4.2)	141 (3.5)	102 (5.8)	< 0.001
Beta-blockers	577 (9.9)	246 (8.6)	231 (13.1)	< 0.001
Antidiabetic agents	224 (3.9)	128 (3.3)	96 (5.5)	< 0.001
Laboratory exam				
Hemoglobin, mg/dL	14.6 ± 15.0	14.6 ± 1.45	14.4 ± 1.61	< 0.001
Total cholesterol, mg/dL	199 ± 36.1	199 ± 35.8	199 ± 36.8	0.940
LDL cholesterol, mg/dL	125 ± 33.0	125 ± 33.2	124 ± 32.5	0.356
HDL cholesterol, mg/dL	52.3 ± 12.9	52.3 ± 12.7	52.1 ± 13.4	0.620
Triglycerides, mg/dL	127 ± 81.4	127 ± 80.6	128 ± 83.2	0.608
Fasting blood glucose, mg/dL	103 ± 23.8	102 ± 23.6	104 ± 24.0	0.055
Creatinine, mg/dL	1.00 ± 0.37	1.02 ± 0.19	1.00 ± 0.60	0.970
eGFR, ml/min/1.73m^2^	78.3 ± 13.4	78.8 ± 13.3	77.2 ± 13.7	< 0.001
Echocardiographic parameter				
LV end-diastolic dimension, mm	48.3 ± 4.03	48.5 ± 3.60	47.6 ± 4.82	< 0.001
LV end-systolic dimension, mm	28.4 ± 3.63	28.6 ± 3.37	28.0 ± 4.13	< 0.001
LV ejection fraction, %	65.1 ± 6.14	65.0 ± 5.94	65.2 ± 6.57	0.270
Left atrial size, mm	36.9 ± 5.16	36.5 ± 4.95	38.0 ± 5.48	< 0.001
IVSd, mm	9.19 ± 1.32	8.77 ± 1.07	10.2 ± 1.32	< 0.001
PWd, mm	9.13 ± 1.32	8.60 ± 1.01	10.3 ± 1.14	< 0.001
RWT, mm	0.38 ± 0.06	0.36 ± 0.04	0.44 ± 0.06	< 0.001
LV mass, g	154 ± 38.1	145 ± 31.0	176 ± 43.7	< 0.001
LV mass index, g/m^2^	87.7 ± 19.5	81.9 ± 14.0	101.1 ± 23.3	< 0.001
PASP, mmHg	27.3 ± 4.78	27.0 ± 4.63	28.1 ± 5.06	< 0.001
E/e’ ratio	9.98 ± 18.1	9.34 ± 2.84	11.4 ± 2.05	0.003

Values are presented as mean ± SD for continuous variables and as numbers (%) for categorical variables

ARB, angiotensin receptor blocker; ACEI, angiotensin-converting enzyme inhibitor; eGFR, estimated glomerular filtration rate; E/e’, the ratio of peak early transmitral inflow velocity to early diastolic velocity of the mitral annulus; HDL, high-density lipoprotein; IVSd, interventricular septum thickness at end-diastole; LDL, low-density lipoprotein; LV, left ventricular; PASP, pulmonary artery systolic pressure; PWd, posterior wall thickness at end-diastole; RWT, relative wall thickness

### Association between abnormal LV geometry and all-cause mortality

During a mean follow-up of 6.2 ± 1.48 years, 84 (1.44%) subjects died in the study population. Of these, 56 subjects were from the normal LV geometry group (1.24%) and 28 were from the abnormal LV geometry group (2.32%). After adjustment for confounding factors, abnormal LV geometry was independently associated with an increased risk of all-cause mortality (adjusted HR 1.64; 95% CI, 1.02–2.67; *p* = 0.040) (Table 2). In the Kaplan–Meier survival analysis, the abnormal LV geometry group showed a worse prognosis than the normal LV geometry group (log-rank *p* < 0.001) (Fig. 1). In the subgroup analysis, the impact of abnormal LV geometry on all-cause mortality was directionally consistent with that in the main analysis (Additional file 1: Table 2).

**Table 2:** Factors associated with all-cause mortality

Variables	Univariate			Multivariate *		
	HR	95% CI	*P* value	HR	95% CI	*P* value
Abnormal LV geometry	2.40	1.563–3.679	< 0.001	1.65	1.022–2.670	0.040
Age, years	1.12	1.093–1.147	< 0.001	1.09	1.054–1.117	< 0.001
Male sex, n (%)	0.99	0.628–1.555	0.957	3.12	1.710–5.709	< 0.001
Body mass index, kg/m^2^	0.94	0.863–1.016	0.113	0.93	0.853–1.014	0.098
Hemoglobin, mg/dL	0.698	0.613–0.795	< 0.001	0.650	0.544–0.776	< 0.001
Total cholesterol, mg/dL	0.99	0.983–0.996	0.001	1.00	0.993–1.006	0.783
eGFR, ml/min/1.73m^2^	0.96	0.947–0.979	< 0.001	0.981	0.963–1.000	0.456

*Adjusted for age, male sex, body mass index, total cholesterol, and eGFR

CI, confidence interval; eGFR, estimated glomerular filtration rate; HR, hazard ratio; LV left ventricular

### Clinical impact of abnormal LV geometry in no or nonobstructive CAD

**Fig. 2:**
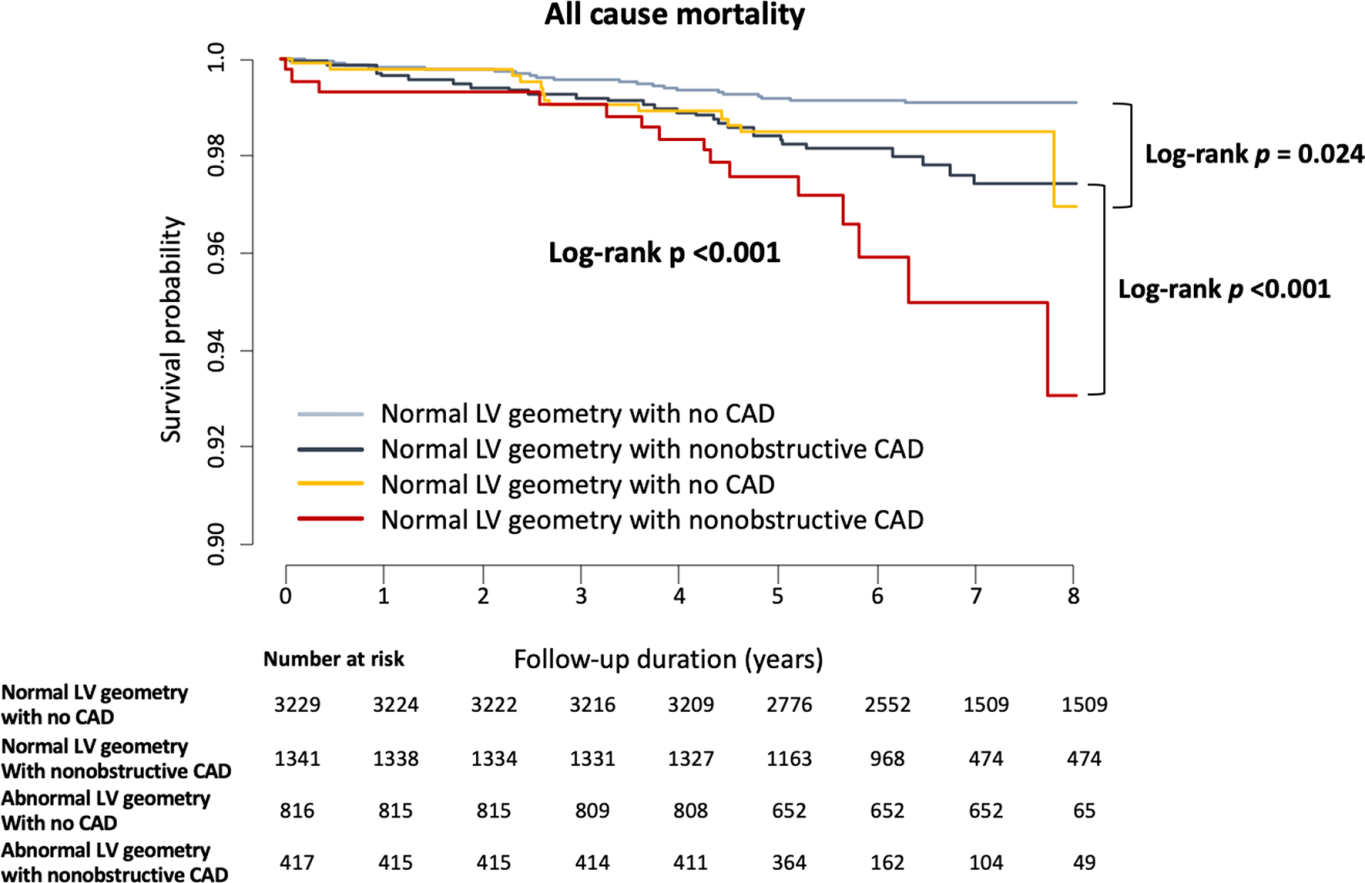
Kaplan–Meier survival curve for subjects with no or nonobstructive CAD on CCTA according to joint categories of LV geometry and CAD status. CAD, coronary artery disease; CCTA, Coronary computed tomography angiography; LV, left ventricular

We classified subjects into four groups according to the joint categories of LV geometry and CAD: 1) normal LV geometry with no CAD, 2) normal LV geometry with nonobstructive CAD, 3) abnormal LV geometry with no CAD, and 4) abnormal LV geometry with nonobstructive CAD. Compared to normal LV geometry without CAD, abnormal LV geometry without CAD (HR 2.38, 95% CI 1.41–4.03, *p* = 0.001) and those with nonobstructive CAD (HR: 4.54, 95% CI: 2.42–8.51, *p* < 0.001) had a higher risk of all-cause mortality (Table 3). In the Kaplan–Meier survival analysis, abnormal LV geometry had worse overall survival in both patients with no CAD (log-rank test, *p* = 0.024) and non-obstructive CAD (log-rank test, *p* < 0.001) (Fig. 2). After adjustment for confounding factors, abnormal LV geometry with nonobstructive CAD remained an independent predictor of all-cause mortality (adjusted HR 2.49; 95% CI, 1.24–4.99; *p* = 0.010) (Table 3).

**Table 3:** Risk of all-cause mortality according to LV geometry and CAD on CCTA

Subgroup	Unadjusted			Adjusted*		
	HR	95% CI	*P* value	HR	95% CI	*P* value
Normal LV geometry with no CAD	Reference	–	Reference			
Normal LV geometry with nonobstructive CAD	2.040	1.055–3.943	0.340	1.687	0.919–3.096	0.091
Abnormal LV geometry with no CAD	2.385	1.412–4.028	0.001	1.656	0.830–3.303	0.153
Abnormal LV geometry with nonobstructive CAD	4.541	2.423–8.511	< 0.001	2.492	1.244–4.990	0.010

*Adjusted for age, male sex, body mass index, hemoglobin, total cholesterol, and estimated glomerular filtration rate

CAD, coronary artery disease; CCTA, Coronary computed tomography angiography; CI, confidence interval; HR, hazard ratio; LV left ventricular

### Association between LV geometry pattern and all-cause mortality

When subjects were stratified into four groups according to LV geometric patterns, each abnormal LV geometric pattern was associated with an increased risk of all-cause mortality compared with normal geometry (log-rank, *p* < 0.001) (Additional file 1: Fig. 2). Eccentric hypertrophy carried the highest risk (HR 3.60, 95% CI 1.91–5.91, *p* < 0.001), followed by concentric hypertrophy (HR 2.34, 95% CI 1.05–5.23, *p* = 0.037), and concentric remodeling (HR 1.92, 95% CI 1.12–3.31, *p* = 0.019). After adjustment, the risk of all-cause mortality was significantly increased only in subjects with eccentric hypertrophy (adjusted HR 2.29; 95% CI, 1.25–4.18; *p* = 0.007) (Additional file 1: Table 3).

### Sensitivity analysis

On repetition of the multivariate Cox proportional regression analysis after further adjusting for medications (beta-blockers and antidiabetic agents) that were significantly associated with all-cause mortality in the univariate analysis, it was found that abnormal LV geometry remained an independent predictor for the primary outcome (HR 1.62; 95% CI, 1.01–2.60; *p* = 0.019).

## Discussion

This study evaluated the prognostic value of abnormal LV geometry estimated by echocardiography in individuals with no or nonobstructive CAD on CCTA. The main findings were as follows: (1) in this cohort with low risk for CAD confirmed by CCTA, echocardiography-determined abnormal LV geometry was associated with an increased risk of all-cause mortality; (2) the prognostic impact of abnormal LV geometry was consistent in both individuals with no CAD and nonobstructive CAD, and 3) eccentric hypertrophy carried the greatest risk of all-cause mortality among different types of abnormal LV geometry.

The presence, extent, and severity of CAD confirmed by CCTA have been well recognized as a strong predictor of adverse outcomes, including death, myocardial infarction, and late revascularization [15, 16]. In this regard, CCTA is widely used as a noninvasive imaging modality for detecting atherosclerotic coronary disease in individuals at low to intermediate risk of cardiovascular disease [17, 18], despite its potential risks, such as overdiagnosis, overtreatment, and unnecessary medical costs [19]. It has been reported that individuals without CAD confirmed by CCTA have a favorable long-term prognosis [20, 21]. In the Coronary CT Angiography Evaluation for Clinical Outcomes: An International Multicenter Registry (CONFIRM) study of 7590 asymptomatic individuals, obstructive CAD also carries a higher risk of mortality and composite outcomes than those with no CAD confirmed by CCTA [19]. Although the prognostic importance of nonobstructive CAD has been underappreciated, several studies have recently shown that nonobstructive CAD confirmed by CCTA is associated with increased mortality risk in various populations [3, 4, 8]. This finding is of clinical importance because effective treatment options, such as statin therapy, are available for patients with nonobstructive CAD, which can improve their prognosis [5].

However, the prevalence of nonobstructive CAD is substantial, and its prognosis can vary among individual patients, making it challenging to apply preventive or therapeutic measures to patients with nonobstructive CAD in general. Specifically, in patients with stable angina, the prevalence of nonobstructive CAD was 67%, with 73% of patients referred to CCTA and 49% among those with invasive coronary angiography [19]. Of note, the prognosis of patients with nonobstructive CAD differs according to several factors based on plaque volume and characteristics [7, 22]. These findings imply that these techniques may be a practical approach allowing the identification of high-risk subgroups of patients with nonobstructive CAD who would benefit from more intensive monitoring and treatment. However, a recent study suggests that whether reporting of CCTA-derived plaque characteristics has clinical implications at this point remains unclear [23]. Furthermore, the assessment of plaque volume and characteristics is challenging to perform, particularly as an integral part of routine clinical practice. Therefore, an easy-to-perform and widely available method to predict outcomes in patients with nonobstructive CAD would be clinically useful in making decisions on the management and follow-up of this heterogeneous group of patients.

In the present study, we demonstrated that abnormal LV geometry determined by echocardiography, which is one of the most frequently used imaging tests in the field of cardiology, was associated with all-cause mortality in individuals with no or nonobstructive CAD confirmed by CCTA. Several lines of evidence support the concept that LV geometry is an imaging marker integrating long-term exposure to both hemodynamic abnormalities (pressure and/or volume overload) and non-hemodynamic factors. Specifically, LV geometry is considered to reflect the severity and chronicity of cardiovascular risk factors, such as hypertension, diabetes mellitus, dyslipidemia, and obesity [24–27], suggesting that LV geometry, as an integrated and cumulative indicator, maybe a better prognosticator than each of the traditional risk factors. It is not surprising that the prognostic role of LV geometry has been extensively investigated in various populations, including patients referred for coronary angiography due to suspected CAD and those following a high-risk myocardial infarction [28]. However, only a few studies have described the association between LV geometry and outcomes in low-risk patients. In this regard, our study showed that individuals with abnormal LV geometry and no or nonobstructive CAD had a dismal prognosis compared with others, highlighting the need for more aggressive monitoring and treatment in this subpopulation.

Given that abnormal LV geometry is a time-integrated indicator of several risk factors that are active in promoting the progression of coronary atherosclerosis and consequently leading to increased adverse clinical outcomes [29], it can be speculated that progression of CAD might be accelerated in individuals with initially no CAD if abnormal LV geometry is present. Indeed, our study demonstrated that during the first 2 years of follow-up, the survival curve of subjects with no CAD but with abnormal LV geometry was nearly identical to that of subjects with no CAD and normal LV geometry; however, the survival curves significantly diverged thereafter. Conversely, all-cause mortality was lower in subjects with no CAD but with abnormal LV geometry than in those with nonobstructive CAD but with normal LV geometry during the first 2 years of follow-up. Nevertheless, the survival curves converged, and the between-group differences were insignificant at the final follow-up. Our study suggested that progression of CAD may be more frequent in individuals with no or nonobstructive CAD who have abnormal LV geometry than in those with normal LV geometry. Further studies with larger sample sizes and longer follow-up durations are needed to confirm our results and speculation.

### Strengths and limitations

The most compelling advantage of our study is that it is a well-constructed, large imaging database study containing both CCTA and echocardiographic images from all participants. Furthermore, our study has an advantage over prior research owing to the inclusion of many Asians in whom the association of LV geometry with prognosis has not been extensively investigated. Specifically, in the landmark Framingham Heart Study (FHS) by Levy et al. [30], participants were predominantly White individuals of Western European descent. Although a more ethnically diverse group of individuals is reflected in the FHS OMNI cohorts, the proportion of the Asian population is only 28% [31], resulting in limited power to draw conclusive results. Additionally, considering the relatively low body mass index in our study population, our findings might be less confounded by overweight or obesity, which is an important confounding factor in studies investigating the association between LV geometry and prognosis [32].

However, several limitations should be considered when interpreting our findings. First, this was a retrospective observational study with inherent limitations, such as unmeasured confounders. Although we adjusted for a set of conventional cardiovascular disease risk factors, residual confounding cannot be completely excluded. Second, the exact clinical indications for CCTA scans were not documented, although most individuals underwent CCTA for the identification of CAD, owing to the presence of chest pain, dyspnea, and cardiovascular risk factors. However, since individuals with obstructive CAD confirmed by CCTA were excluded from our study, the difference in the clinical indications for CCTA scans might not substantially affect the results of this study. Third, since we focused on all-cause mortality as the primary outcome, data on specific causes of death were not available. In observational studies, all-cause mortality is generally considered a more robust and unbiased outcome than disease-specific mortality. However, data on the specific cause of death might have strengthened the association between LV geometry and cardiovascular prognosis in subjects with no or nonobstructive CAD. Fourth, there remains a possibility that certain conditions affecting both LV structure and mortality were not fully ruled out. To minimize this concern, we excluded individuals with cardiomyopathy and significant valvular heart disease from the analyses. Fifth, as Korean individuals were exclusively included in the present study, it is uncertain whether our findings can be generalized to other populations. Lastly, since there are no data on noninvasive stress testing, we could not entirely exclude the possibility of myocardial ischemia, which is clearly a confounding factor related to mortality.

## Conclusions

Abnormal LV geometry carried a worse prognosis than normal LV geometry in individuals with no or nonobstructive CAD confirmed by CCTA. These findings suggest that the use of echocardiography for LV geometry assessment has the potential to be a clinically useful and practical approach for risk stratification in this population.

## Supplementary Information

**Additional file 1: **Supplementary tables and figures.

## Data Availability

The datasets generated and/or analyzed during the current study are not publicly available because of data collected from multicenter, but are available from the corresponding author upon reasonable request.

## Abbreviations

CAD: Coronary artery disease
CCTA: Coronary computed tomography angiography
CI: Confidence interval
eGFR: Estimated glomerular filtration rate
HR: Hazard ratio
LV: Left ventricular
LVMI: Left ventricular mass index
LVEDD: Left ventricular end-diastolic dimension
RWT: Relative wall thickness
