# Supplementary material for: Prognostic implications of left ventricular mass-geometry in patients with no or nonobstructive coronary artery disease
Source: BMC Cardiovasc Disord. 2021 Apr 15;21:187. doi: 10.1186/s12872-021-02005-6 (PMC8051046; doi:10.1186/s12872-021-02005-6)
Supplement: Supplementary file 1 — Additional file 1: Supplementary tables and figures. [file 12872_2021_2005_MOESM1_ESM.docx]

Supplementary materials

Prognostic Implications of Left Ventricular Mass-Geometry in Patients with No or Nonobstructive Coronary Artery Disease

**Short title:** Left ventricular geometry and no or nonobstructive coronary artery disease

You-Jung Choi, MD^1, 2^, Jun-Bean Park, MD, PhD^1, 2*^, Chan Soon Park, MD^1,3^, Inchang Hwang, MD ^1, 4^, Yeonyee E. Yoon, MD, PhD^1, 4^, Seung-Pyo Lee, MD, PhD ^1,2^, Hyung-Kwan Kim, MD, PhD ^1, 2^, Yong-Jin Kim, MD, PhD ^1, 2^, Goo-Yeong Cho, MD, PhD ^1, 4^, and Dae-Won Sohn, MD, PhD ^1, 2^

^1^Department of Internal Medicine, Seoul National University College of Medicine, Seoul, Republic of Korea

^2^Cardiovascular Center, Seoul National University Hospital, Seoul, Republic of Korea

^3^Graduate school of Medical Science and Engineering, Korea Advanced Institute of Science and Technology, Daejeon, Republic of Korea

^4^Cardiovascular Center, Seoul National University Bundang Hospital, Seongnam, Gyeonggi-do, Republic of Korea

**Corresponding author:**

**Jun-Bean Park, MD, PhD**

Associate Professor, Division of Cardiology, Department of Internal Medicine, Seoul National University College of Medicine/Cardiovascular Center, Seoul National University Hospital

101 Daehak-ro, Jongno-gu, Seoul, 03080, Korea

Telephone: +82-2-2072-4190; Fax: +82-2-2072-4922; E-mail: nanumy1@gmail.com

**
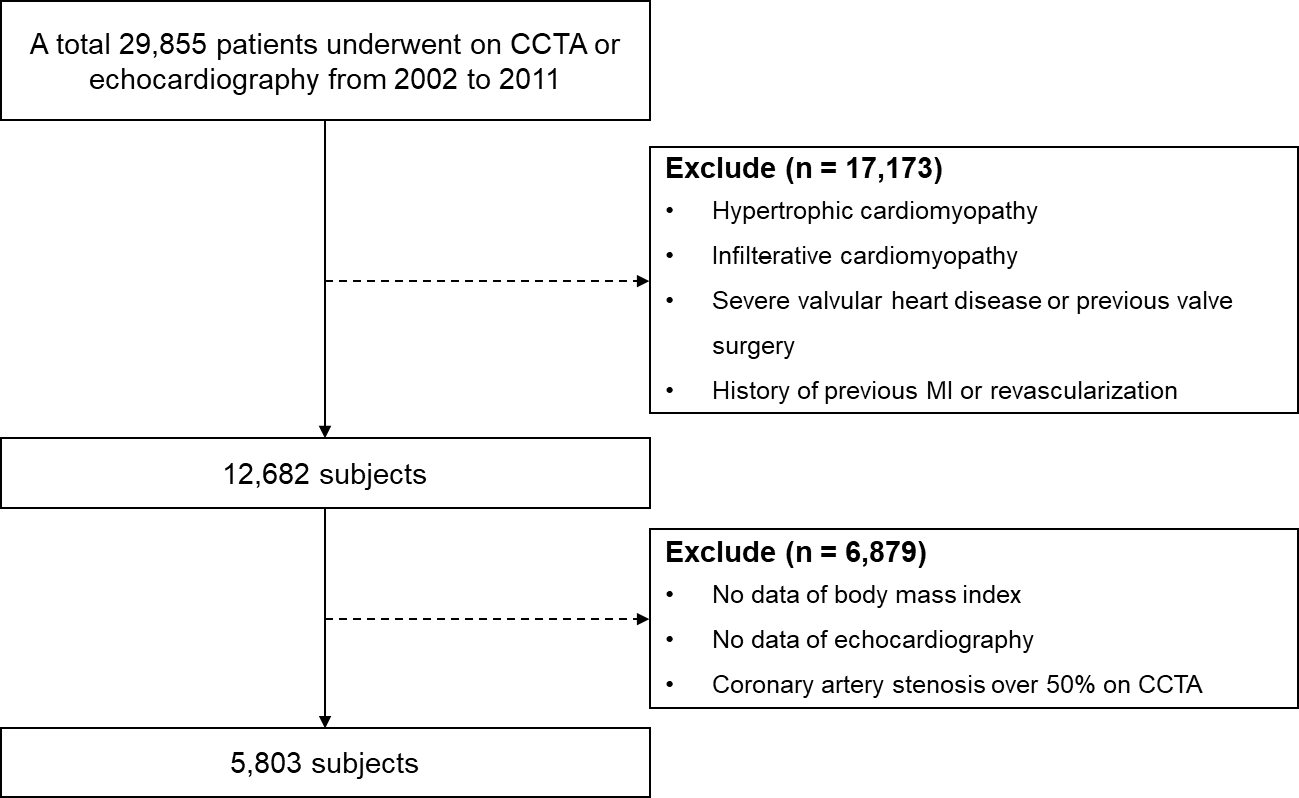
**

**Supplementary Figure 1.** Flow chart of the study population

CCTA, coronary computed tomographic angiography; MI, myocardial infarction.

**
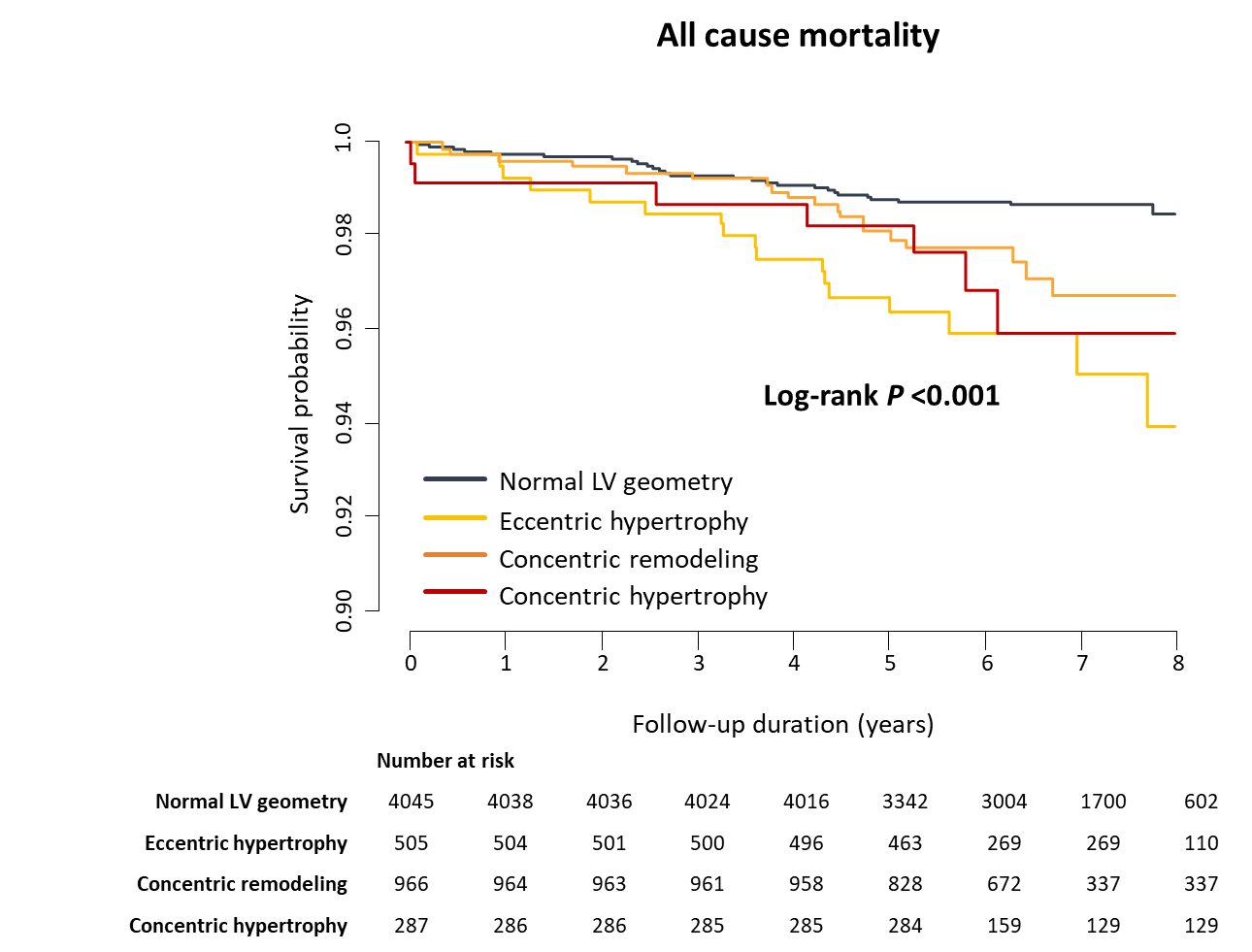
**

**Supplementary Figure 2.** Kaplan-Meier survival curve for subjects with no nor nonobstructive CAD on CCTA according to LV geometric patterns**.**

Survival curves were compared with the log-rank test.

Abnormal geometry indicates LVMI >95 g/m^2^ in women and >115 g/m^2^ in men, and the cutoff for abnormal RWT was >0.42 in both women and men. Subjects were classified into four groups as follows: normal geometry (both normal LVMI and RWT), concentric remodeling (normal LVMI and increased RWT), eccentric hypertrophy (increased LVMI and normal RWT), and concentric hypertrophy (both increased LVMI and RWT).

CAD, coronary artery disease; CCTA, coronary computed tomography angiography; LV, left ventricular; LVMI, left ventricular mass index; RWT, relative wall thickness.

**Supplementary Table 1.** Baseline characteristics according to left ventricular geometric pattern

| **Values** | **Normal geometry**  **(n=4,045)** | **Eccentric hypertrophy**  **(n=966)** | **Concentric remodeling**  **(n=505)** | **Concentric hypertrophy**  **(n=287)** | ***P* value** |
| --- | --- | --- | --- | --- | --- |
| Age, years | 55.7 ± 8.68 | 60.9 ± 8.79 | 56.8 ± 8.68 | 60.3 ± 9.04 | <0.001 |
| Male sex, n (%) | 2,816 (69.6) | 159 (31.5) | 752 (77.8) | 157 (54.7) | <0.001 |
| Body surface area, m^2^ | 1.76 ± 0.17 | 1.65 ± 0.16 | 1.80 ± 0.18 | 1.73 ± 0.19 | <0.001 |
| Body mass index, kg/m^2^ | 24.3 ± 2.77 | 24.1 ± 3.04 | 24.9 ± 2.93 | 24.9 ± 3.00 | <0.001 |
| Laboratory exam |  |  |  |  |  |
| Hemoglobin, mg/dL | 1.46 ± 1.45 | 13.+ ± 1.53 | 14.9 ± 1.45 | 14.1 ± 1.62 | <0.001 |
| Total cholesterol, mg/dL | 199 ± 35.8 | 197 ± 35.8 | 200 ± 37.4 | 197 ± 36.9 | 0.409 |
| LDL cholesterol, mg/dL | 127 ± 80.6 | 119 ± 87.2 | 134 ± 85.3 | 121 ± 65.2 | 0.007 |
| HDL cholesterol, mg/dL | 125 ± 33.2 | 125 ± 31.6 | 126 ± 33.1 | 121 ± 31.8 | 0.037 |
| Triglycerides, mg/dL | 52.3 ± 12.7 | 53.6 ± 13.6 | 50.9 ± 12.8 | 53.6 ± 14.6 | <0.001 |
| Fasting blood glucose, mg/dL | 102 ± 23.6 | 103 ± 22.0 | 104 ± 22.6 | 107.5 ± 31.1 | 0.004 |
| Creatinine, mg/dL | 1.00 ± 0.19 | 0.91 ± 0.39 | 1.03 ± 0.17 | 1.07 ± 1.37 | <0.001 |
| eGRF, ml/min/1.73m^2^ | 78.8 ± 13.3 | 78.1 ± 14.5 | 77.4 ± 12.8 | 74.8 ± 14.8 | <0.001 |
| Echocardiographic parameter |  |  |  |  |  |
| LV end-diastolic dimension, mm | 48.5 ± 3.60 | 52.2 ± 3.46 | 44.8 ± 3.42 | 49.1 ± 4.09 | <0.001 |
| LV end-systolic dimension, mm | 38.6 ± 3.38 | 30.7 ± 4.26 | 26.4 ± 3.07 | 28.8 ± 4.28 | <0.001 |
| LV ejection fraction, % | 65.0 ± 5.94 | 65.2 ± 7.17 | 65.2 ± 6.08 | 65.3 ± 7.04 | 0.714 |
| E/e’ ratio | 9.33 ± 2.84 | 14.7 ± 57.9 | 9.52 ± 3.07 | 11.8 ± 9.60 | <0.001 |
| PASP, mmHg | 27.0 ± 4.63 | 29.0 ± 5.65 | 27.6 ± 4.81 | 27.5 ± 4.15 | <0.001 |

Values presents as mean ± SD for continuous variables, and as the number (%) for categorical variables.

eGFR, estimated glomerular filtration rate; E/e’, the ratio of peak early transmitral inflow velocity to early diastolic velocity of the mitral annulus; HDL, high-density lipoprotein; LA, left atrium; LDL, low-density lipoprotein; LV, left ventricular; PASP, pulmonary artery systolic pressure.

**Supplementary Table 2.** Subgroups analysis

| **Subgroup** | **Univariate analysis** | | | **Multivariate**^a^ | | |
| --- | --- | --- | --- | --- | --- | --- |
|  | **HR** | **95% CI** | ***P* value** | **HR** | **95% CI** | ***P* value** |
| Age |  |  |  |  |  |  |
| <65-year-old | 2.83 | 1.329–6.016 | 0.007 | 2.59 | 1.175–5.690 | 0.018 |
| ≥65-year-old | 1.45 | 0.817–2.568 | 0.204 | 1.57 | 0.831–2.961 | 0.164 |
| Sex |  |  |  |  |  |  |
| Male | 1.98 | 1.163–3.353 | 0.012 | 1.38 | 0.768–2.478 | 0.281 |
| Female | 3.72 | 1.681–8.225 | 0.001 | 2.84 | 1.181–6.849 | 0.019 |
| eGFR |  |  |  |  |  |  |
| eGFR <60 ml/min/1.73m^2^ | 2.86 | 1.140–7.172 | 0.025 | 2.08 | 0.790–5.475 | 0.138 |
| eGFR ≥60 ml/min/1.73m^2^ | 2.04 | 1.230–3.369 | 0.006 | 1.63 | 0.946–2.823 | 0.079 |
| LV systolic function |  |  |  |  |  |  |
| Ejection fraction <60 % | 3.21 | 1.581–6.499 | 0.001 | 2.13 | 0.918–4.925 | 0.078 |
| Ejection fraction ≥60 % | 2.14 | 1.213–3.765 | 0.009 | 1.71 | 0.935–3.122 | 0.082 |

^a^Adjusted for age, male, body mass index, total cholesterol, and eGFR.

CI, confidence interval; eGFR, estimated glomerular filtration rate; HR, hazard ratio; LV, left ventricular.

**Supplementary Table 3. Risk of all-cause mortality according to left ventricular geometric pattern**

| **Variables** | **Total** | **Event** | **Unadjusted** | | |  | **Age- and sex-adjusted** | | |
| --- | --- | --- | --- | --- | --- | --- | --- | --- | --- |
|  |  |  | **HR** | **95% CI** | ***P* value** |  | **HR** | **95% CI** | ***P* value** |
| **Normal LV geometry** | 4,405 | 41 | 1 | (reference) |  |  | 1 | (reference) |  |
| **Abnormal LV geometry**^a^ | 1,788 | 43 | 2.40 | 1.563–3.679 | <0.001 |  | 1.79 | 1.157–2.773 | 0.009 |
| Eccentric hypertrophy | 966 | 17 | 3.60 | 1.909–5.914 | <0.001 |  | 2.29 | 1.249–4.179 | 0.007 |
| Concentric remodeling | 505 | 19 | 1.92 | 1.115–3.310 | 0.019 |  | 1.64 | 0.951–2.834 | 0.075 |
| Concentric hypertrophy | 287 | 7 | 2.34 | 1.051–5.226 | 0.037 |  | 1.50 | 0.651–3.318 | 0.353 |

^a^Abnormal geometry indicates LV mass index >95 g/m^2^ in women and >115 g/m^2^ in men and the cutoff for abnormal relative wall thickness was >0.42 in both women and men.

CI, confidence interval; HR, hazard ratio; LV, left ventricular.
